# Impact of right ventricular-to-pulmonary artery coupling on remodeling and outcome in patients undergoing transcatheter edge-to-edge mitral valve repair

**DOI:** 10.1007/s00392-023-02318-w

**Published:** 2023-10-23

**Authors:** Matthias Koschutnik, Carolina Donà, Christian Nitsche, Andreas A. Kammerlander, Varius Dannenberg, Christina Brunner, Sophia Koschatko, Katharina Mascherbauer, Gregor Heitzinger, Kseniya Halavina, Georg Spinka, Max-Paul Winter, Martin Hülsmann, Philipp E. Bartko, Christian Hengstenberg, Julia Mascherbauer, Georg Goliasch

**Affiliations:** 1 Department of Internal Medicine II, Division of Cardiology, Medical University of Vienna, Waehringer Guertel 18-20, 1090 Vienna, Austria; 2 Department of Internal Medicine 3, University Hospital St. Poelten, Karl Landsteiner University of Health Sciences, Krems, Austria

**Keywords:** RV–PA coupling, RV function, TEER, Mitral regurgitation, Outcome

## Abstract

**Background:**

Right ventricular-to-pulmonary artery (RV–PA) coupling has recently been shown to be associated with outcome in valvular heart disease. However, longitudinal data on RV dysfunction and reverse cardiac remodeling in patients following transcatheter edge-to-edge mitral valve repair (M-TEER) are scarce.

**Methods:**

Consecutive patients with primary as well as secondary mitral regurgitation (MR) were prospectively enrolled and had comprehensive echocardiographic and invasive hemodynamic assessment at baseline. Kaplan–Meier estimates and multivariable Cox-regression analyses were performed, using a composite endpoint of heart failure hospitalization and death.

**Results:**

Between April 2018 and January 2021, 156 patients (median 78 y/o, 55% female, EuroSCORE II: 6.9%) underwent M-TEER. On presentation, 64% showed impaired RV–PA coupling defined as tricuspid annular plane systolic excursion to pulmonary artery systolic pressure (TAPSE/PASP) ratio < 0.36. Event-free survival rates at 2 years were significantly lower among patients with impaired coupling (57 vs. 82%, *p* < 0.001), both in patients with primary (64 vs. 91%, *p* = 0.009) and secondary MR (54 vs. 76%, *p* = 0.026). On multivariable Cox-regression analyses adjusted for baseline, imaging, hemodynamic, and procedural data, TAPSE/PASP ratio < 0.36 was independently associated with outcome (adj.HR 2.74, 95% CI 1.17–6.43, *p* = 0.021).

At 1-year follow-up, RV–PA coupling improved (TAPSE: ∆ + 3 mm, PASP: ∆ − 10 mmHg, *p* for both < 0.001), alongside with a reduction in tricuspid regurgitation (TR) severity (grade ≥ II: 77–54%, *p* < 0.001).

**Conclusions:**

TAPSE/PASP ratio was associated with outcome in patients undergoing M-TEER for primary as well as secondary MR. RV–PA coupling, alongside with TR severity, improved after M-TEER and might thus provide prognostic information in addition to established markers of poor outcome.

**Graphical abstract:**

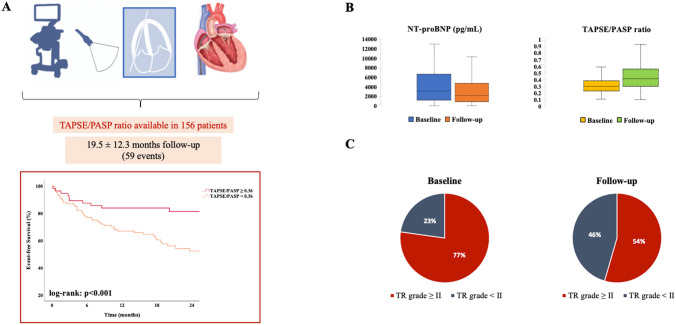

**Supplementary Information:**

The online version contains supplementary material available at 10.1007/s00392-023-02318-w.

## Introduction

Transcatheter edge-to-edge mitral valve repair (M-TEER) has recently been introduced as treatment option for patients with significant mitral regurgitation (MR) [1, 2]. Based on emerging evidence of favorable long-term durability, M-TEER is increasingly used in clinical practice [3, 4]. However, pre-procedural risk assessment remains challenging and longitudinal data on reverse cardiac remodeling and outcome are limited.

Previous studies have highlighted the prognostic significance of right ventricular-to-pulmonary artery (RV–PA) coupling, as a more robust measurement of RV dysfunction, in patients with heart failure [5], pulmonary hypertension [6], and/or valvular heart disease [7]. In a retrospective analysis of the EuroSMR registry, Karam et al. [8] have recently demonstrated that impaired RV–PA coupling, defined as tricuspid annular plane systolic excursion (TAPSE)-to-PA systolic pressure (PASP) ratio, was an important predictor of mortality in patients undergoing M-TEER for secondary MR. However, according to the newly issued ACC/AHA guidelines [2], M-TEER is not only considered for patients with secondary MR and poor left-ventricular (LV) systolic function, but also received a IIa recommendation for primary MR. Yet, optimal patient selection is crucial, especially in light of conflicting results from large randomized trials [4, 9].

Accordingly, for the first time, our prospective all-comer study of consecutive patients undergoing both comprehensive echocardiographic and invasive hemodynamic assessment prior to M-TEER aimed to investigate the impact of RV–PA coupling on outcome and reverse RV remodeling in patients with primary as well as secondary MR.

## Methods

### Study design

This prospective, observational study was conducted at the Medical University of Vienna, Austria, a university-affiliated tertiary care center with a multimodality imaging laboratory and a high-volume cardiac catheterization unit. Consecutive patients with significant MR scheduled for M-TEER were recruited. All cases were discussed individually by our local multidisciplinary Heart Team. The investigation conforms to the principles outlined in the Declaration of Helsinki, and the study protocol was approved by our Institutional Review Board (identifier: EK 1881/2012, amended version 01/2021). Written informed consent was obtained in all patients prior to study enrollment.

### Echocardiography

Comprehensive echocardiographic assessments, including transesophageal echocardiography (TEE), were performed by board-certified cardiologists using high-end scanners (e.g., Vivid E95, GE Healthcare, Chicago, IL, USA). Cardiac chamber size was assessed according to current recommendations [10]. LVEF was calculated using the biplane Simpson’s method. RV systolic function was assessed by TAPSE, fractional area change (FAC), systolic movement of the RV lateral wall using tissue Doppler imaging (S’), and 2D speckle tracking echocardiography, based on the current guidelines [11]. RV dysfunction was defined by TAPSE < 17 mm, RV FAC < 35%, S’ < 9.5 cm/s, and RV free-lateral-wall strain > − 20% (less negative values indicate impaired function). These cut-off values were chosen according to previous studies [12, 13] and current recommendations [10, 14]. PASP was calculated by adding the peak tricuspid regurgitation (TR) systolic gradient to the estimated central venous pressure. RV–PA coupling was assessed using the TAPSE/PASP ratio. Valvular heart disease was quantified using an integrated approach as recommended in the respective guidelines [15, 16]. Severity of MR was determined using morphological criteria and jet direction (myxomatous degeneration, leaflet prolapse/flail), as well as quantification by vena contracta width, estimated regurgitant volume, and the effective regurgitant orifice area. In accordance with the previously published literature [3, 16], we applied a scale ranging from 1 to 4 in order to define MR severity: grade 1 indicates “mild”, 2 “moderate”, 3 “moderate-to-severe”, and 4 refers to “severe” MR. Sonographers were blinded to procedural and outcome data.

### Invasive hemodynamics

Invasive hemodynamic assessment was performed routinely in study participants immediately prior to M-TEER. Hemodynamic measurements were performed using a 7F Swan-Ganz catheter (Edwards Lifesciences GmbH, Austria) via a femoral access. Pressures were documented as average of eight measurements over eight consecutive heart cycles using CathCorLX (Siemens AG, Berlin and Munich, Germany). In addition to PA wedge pressure (PAWP), the systolic (sPAP), diastolic (dPAP) and mean PA (mPAP) pressures were documented. TAPSE/sPAP_inv_ ratio was calculated using the invasively measured sPAP. Cardiac output (CO) was measured by Fick’s method or thermodilution. If both were available, Fick’s method was preferred. Furthermore, the transpulmonary gradient (TPG) and diastolic pulmonary vascular pressure gradient (DPG) were calculated according to the current guidelines [17]. TPG was computed by subtracting PAWP from mPAP; DPG was calculated as the difference between dPAP and PAWP during pull-back; pulmonary vascular resistance (PVR) was calculated by dividing TPG by CO.

### TEER procedures

All procedures were performed under general anesthesia with TEE and fluoroscopic guidance [18]. In brief, the edge-to-edge mitral repair system (MitraClip™ NTR, XTR, or PASCAL™) was introduced through the femoral vein and advanced to the MV by crossing the inter-atrial septum. Up to three edge-to-edge devices were placed into the MV to maximally reduce MR. Concomitant transcatheter edge-to-edge tricuspid valve repair (T-TEER) was performed in patients with significant TR by discretion of our Heart Team.

### Outcome measures

Patients were prospectively followed after M-TEER in a dedicated outpatient clinic at 3 months, 12 months, and yearly thereafter. The primary outcome measure was a combined endpoint consisting of HF hospitalization (HFH) and death. HFH was defined as inpatient admission with clinical signs and symptoms of HF and requirement for intravenous diuretic treatment. Endpoints were ascertained by follow-up visits, state-wide electronic hospital charts, and direct patient phone calls. Mortality data were obtained via the National Registry of Deaths (Statistics Austria). The internal adjudication committee, consisting of CH and JM, blinded to echocardiography and procedural data, confirmed all endpoints.

### Statistical analysis

Continuous data are presented as median (interquartile range [IQR]), categorical variables as total numbers and percentages, respectively. Comparisons between groups were performed using either Chi-squared or Fisher’s exact tests for categorical variables or Wilcoxon rank-sum tests for continuous variables, as appropriate. For correlation analyses, Spearman’s coefficients were used. In line with the previous studies [19, 20], the optimal cut-off for impaired RV–PA coupling was defined as TAPSE/PASP ratio < 0.36. Kaplan–Meier curves were plotted and the Log-rank test was used to estimate differences between survival curves. Cox-regression models were calculated to investigate the association between RV–PA coupling and the composite endpoint of HFH and death. In addition to crude analyses, we pre-defined three multivariable models: Model (A) adjusted for the EuroSCORE II, Model (B) adjusted for the EuroSCORE II and logarithmized NT-proBNP levels, and Model (C) adjusted for clinical and imaging variables with a significant impact on outcome using a stepwise multivariable model, which included all variables with a significant influence on an univariable level. To account for potential bias, we then repeated outcome analysis in patients who underwent isolated M-TEER in absence of concomitant T-TEER. Comparisons between baseline and follow-up measures were performed using either Chi-squared or Fisher’s exact tests or Wilcoxon signed-rank tests including and excluding patients who had concomitant T-TEER, respectively. A two-sided *p* value < 0.05 was considered statistically significant. All analyses were performed using SPSS 27 (IBM SPSS, USA).

## Results

### Baseline characteristics

In total, 163 consecutive patients scheduled for M-TEER were screened between April 2018 and January 2021. TAPSE/PASP ratio was available in 156 (96%) patients, who were included in the final analysis. Baseline characteristics and concomitant medication are summarized in Table 1. Impaired RV–PA coupling was present in 100 (64%) individuals. Compared to patients with normal coupling, TAPSE/PASP ratio was significantly associated with a higher EuroSCORE II (8.9 vs. 5.0%, *p* = 0.001), elevated NT-proBNP levels (4149 vs. 2612 pg/mL, *p* = 0.008), and impaired renal function (Creatinine: 1.3 vs. 1.2 mg/dL, estimated glomerular filtration rate [eGFR]: 47 vs. 52 mL/min/1.73m^2^, *p* for both < 0.041) at baseline. Impaired coupling was further associated with coronary artery disease (59 vs. 41%, *p* = 0.031), previous coronary artery bypass grafting (24 vs. 11%, *p* = 0.043), as well as pacemaker implantation (38 vs. 21%, *p* = 0.033).

**Table 1:** Baseline characteristics stratified for RV–PA coupling

	Overall population (*n* = 156)	Impaired RV–PA coupling (*n* = 100)	Normal RV–PA coupling (*n* = 56)	*p* value
Clinical parameters				
Age (years)	78 [72–83]	78 [72– 83]	79 [73–83]	0.720
Female sex, % [*n*]	55 [85]	53 [53]	57 [32]	0.618
Body mass index (kg/m^2^)	24.5 [22.5–28.5]	24.2 [22.4–28.6]	25.5 [22.6–28.5]	0.381
EuroSCORE II (%)	6.9 [3.9–10.8]	8.9 [4.0–14.4]	5.0 [2.5–8.5]	**0.001**
NYHA functional class ≥ III, % [*n*]	89 [138]	86 [86]	93 [52]	0.296
NT-proBNP (pg/mL)	3254 [1380–6827]	4149 [1677–9180]	2612 [831–5237]	**0.008**
Creatinine (mg/dL)	1.3 [1.0–1.8]	1.3 [1.1–1.9]	1.2 [0.9–1.7]	**0.041**
eGFR (mL/min/1.73m^2^)	48 [35–66]	47 [33–63]	52 [38–76]	**0.031**
Co-morbidities				
Coronary artery disease, % [*n*]	53 [82]	59 [59]	41 [23]	**0.031**
Myocardial infarction, % [*n*]	22 [35]	23 [23]	21 [12]	0.821
Percutaneous coronary intervention, % [*n*]	35 [54]	35 [35]	34 [19]	0.893
Coronary artery bypass grafting, % [*n*]	19 [30]	24 [24]	11 [6]	**0.043**
Previous valve surgery, % [*n*]	17 [27]	20 [20]	13 [7]	0.235
Atrial fibrillation, % [*n*]	67 [107]	69 [169]	68 [38]	0.883
Previous pacemaker implantation, % [*n*]	32 [50]	38 [38]	21 [12]	**0.033**
Arterial hypertension, % [*n*]	94 [147]	93 [93]	96 [54]	0.491
Diabetes mellitus type II, % [*n*]	28 [43]	31 [31]	21 [12]	0.199
Hyperlipidemia, % [*n*]	66 [103]	64 [64]	70 [39]	0.475
Previous stroke, % [*n*]	7 [11]	10 [10]	2 [1]	0.099
Cerebral artery disease, % [*n*]	14 [21]	13 [13]	14 [8]	0.821
Peripheral artery disease, % [*n*]	12 [19]	16 [16]	5 [3]	0.072
COPD, % [*n*]	21 [32]	20 [20]	21 [12]	0.832
Concomitant medication				
Beta blockers, % [*n*]	74 [116]	79 [79]	66 [37]	0.076
ACE inhibitors, % [*n*]	30 [46]	26 [26]	36 [20]	0.202
Angiotensin receptor blockers, % [*n*]	21 [33]	19 [19]	25 [14]	0.379
ARNIs, % [*n*]	15 [24]	18 [18]	11 [6]	0.226
SGLT-2 inhibitors, % [*n*]	6 [10]	6 [6]	7 [4]	0.747
Calcium channel blockers, % [*n*]	12 [18]	12 [12]	11 [6]	0.809
Loop diuretics, % [*n*]/daily dose (mg)	71 [110]/40 [40–80]	75 [75]/40 [40–80]	63 [35]/40 [20–80]	0.100
Thiazide diuretics, % [*n*]/daily dose (mg)	13 [20]/32.5 [12.5–40]	14 [14]/40 [25–40]	11 [6]/12.5 [12.5–19.4]	0.556
Spironolactone, % [*n*]/daily dose (mg)	58 [90]/50 [25–50]	61 [61]/50 [25–50]	52 [29]/50 [25–50]	0.264
Oral anticoagulants, % [*n*]	62 [96]	63 [63]	59 [33]	0.616
Vitamin-K-Antagonists, % [*n*]	11 [17]	11 [11]	11 [6]	0.956
Statins, % [*n*]	60 [94]	57 [57]	66 [37]	0.267

Values are given as median [IQR] or % [*n*]

A two-sided *p* value < 0.05 was considered statistically significant (in bold)

*RV* right ventricular, *PA* pulmonary artery, *NYHA* New York Heart Association, *NT-proBNP* N-terminal prohormone of brain natriuretic peptide, *eGFR* estimated glomerular filtration rate, *COPD* chronic obstructive pulmonary disease, *ACE* angiotensin converting enzyme, *ARNI* angiotensin receptor neprilysin inhibitor, *SGLT-2* sodium glucose transporter 2

### Imaging data and invasive hemodynamics

Table 2 summarizes echocardiographic and Table 3 summarizes hemodynamic data at baseline. Patients with impaired RV–PA coupling showed significantly lower RV FAC (34.9 vs. 40.5%), S’ (9 vs. 10 cm/s), and higher RV global longitudinal strain (− 17 vs. − 20%), indicating worse RV function compared to patients with normal coupling (*p* for all < 0.001). With regards to RV free-lateral-wall strain, no significant differences between both groups could be found (− 18 vs. − 21%, *p* = 0.074). We were able to measure all 5 echocardiographic RV function parameters in 134 (86%) individuals. Inter-observer variability showed moderate-to-good agreement in a subset of 20 patients (ICC for all > 0.704, Supplemental Table 1). Patients with impaired RV–PA coupling had a more dilated RV (end-diastolic diameter: 39 vs. 36 mm, *p* < 0.001), alongside with advanced TR severity (grade ≥ II: 92 vs. 68%, *p* < 0.001). Concomitant T-TEER was performed in 36 (23%) patients. Baseline TAPSE/PASP ratio < 0.36 was similar among patients with and without additional T-TEER (72 vs. 62%, *p* = 0.247).

With regards to hemodynamic assessment, mPAP (25 vs. 19 mmHg), sPAP (37 vs. 34 mmHg), and PAWP (14 vs. 10 mmHg) were significantly higher in patients with impaired RV–PA coupling (*p* for all < 0.047). We found only modest correlation between conventional TAPSE/PASP ratio and invasively measured sPAP (TAPSE/sPAP_inv_ ratio: *r* = 0.5, *p* < 0.001). Nevertheless, among patients with impaired coupling on echocardiography, TAPSE/sPAP_inv_ ratio remained significantly lower, when compared to normal coupling (0.40 vs. 0.57, *p* < 0.001).

**Table 2:** Baseline echocardiography and procedural data stratified for RV–PA coupling

	Overall population (*n* = 156)	Impaired RV–PA coupling (*n* = 100)	Normal RV–PA coupling (*n* = 56)	*p* value
Echocardiographic parameters				
LV end-diastolic diameter (mm)	50 [44–56]	51 [44–57]	49 [43–55]	0.647
RV end-diastolic diameter (mm)	38 [32–42]	39 [35–43]	36 [30–39]	** < 0.001**
Interventricular septum (mm)	13 [11–14]	12 [11–14]	13 [12–14]	0.145
Aorta ascendens (mm)	34 [32–37]	34 [31–36]	34 [32–37]	0.161
LV ejection fraction (%)	47 [31–54]	47 [30–54]	52 [35–54]	0.425
TAPSE (mm)	16 [15–19]	16 [15–17]	19 [17–22]	** < 0.001**
RV fractional area change (%)	37.1 [30.0–43.8]	34.9 [27.1–41.7]	40.5 [36.0–46.0]	** < 0.001**
S‘ (cm/s)	10 [8–11]	9 [8–11]	10 [10–12]	** < 0.001**
RV free-lateral-wall strain (%)	− 19 [− 21 to − 15]	− 18 [− 21 to − 15]	− 21 [− 22 to − 15]	0.074
RV global longitudinal strain (%)	− 18 [− 20 to − 15]	− 17 [− 19 to − 13]	− 20 [− 23 to − 17]	** < 0.001**
PASP (mmHg)	57 [48–69]	64 [55–74]	43 [36–50]	** < 0.001**
TAPSE/PASP ratio	0.30 [0.23–0.39]	0.25 [0.21–0.29]	0.44 [0.38–0.50]	** < 0.001**
MR ≥ moderate, % [*n*]	99 [155]	100 [100]	98 [55]	0.359
MR etiology				0.313
Primary, % [*n*]	36 [56]	33 [33]	41 [23]	
Secondary, % [*n*]	64 [100]	67 [67]	59 [33]	
TR ≥ moderate, % [*n*]	83 [130]	92 [92]	68 [38]	** < 0.001**
Procedural data				
No. of clips implanted				
1, 2, or 3 (%)	52, 45, 3	45, 51, 4	64, 34, 2	
NTR, XTR, or PASCAL (%)	59, 36, 5	62, 33, 4	53, 42, 5	
Concomitant T-TEER procedure, % [*n*]	23 (36)	26 (26)	18 (10)	0.247
MR post-procedural < moderate, % [*n*]	88 (137)	85 (85)	93 (52)	0.204
MV meanPG post-procedural (mmHg)	3 [2–4]	3 [2–4]	3 [2–4]	**0.023**

Values are given as median [IQR] or % [*n*]

A two-sided *p* value < 0.05 was considered statistically significant (in bold)

*RV* right ventricular, *PA* pulmonary artery, *LV* left ventricular, *TAPSE* tricuspid annular plane systolic excursion, *PASP* pulmonary artery systolic pressure, *MR* mitral regurgitation, *TR* tricuspid regurgitation, *T-TEER* transcatheter edge-to-edge tricuspid valve repair, *MV* mitral valve, *meanPG* mean pressure gradient

**Table 3:** Hemodynamic assessment stratified for RV–PA coupling

	Overall population (*n* = 156)	Impaired RV–PA coupling (*n* = 100)	Normal RV–PA coupling (*n* = 56)	*p* value
Mean PAP (mmHg)	22 [17–32]	25 [19–34]	19 [14–30]	**0.027**
Systolic PAP (mmHg)	35 [28–48]	37 [30–51]	34 [25–43]	**0.047**
Diastolic PAP (mmHg)	13 [9–20]	13 [10–21]	12 [8–18]	0.396
TAPSE/sPAP_inv_ ratio	0.46 [0.35–0.66]	0.40 [0.29–0.57]	0.57 [0.39–0.79]	** < 0.001**
PAWP mean (mmHg)	13 [8–20]	14 [10–21]	10 [6–19]	**0.016**
DPG (mmHg)*	3 [1–5]	3 [1–6]	2 [1–5]	0.644
TPG (mmHg)*	10 [7–13]	11 [7–13]	10 [7–13]	0.414
LAP mean (mmHg)	10 [6–15]	10 [7–16]	9 [5–14]	0.063
RAP mean (mmHg)	6 [3–11]	8 [3–11]	4 [2–9]	0.158
PP (mmHg)	22 [16–28]	25 [18–31]	20 [14–27]	**0.026**
PVR (dyne/s/cm^5^)	224 [152–300]	220 [156–334]	232 [147–290]	0.660
PAC (mL/mmHg)	2.8 [1.8–4.1]	2.4 [1.6–4.1]	3.4 [2.2–4.4]	0.056
CO (L/min)	4.1 [3.5–5.3]	4.3 [3.5–5.3]	3.9 [3.4–5.2]	0.443
CI (L/min/m^2^)	2.2 [1.9–2.7]	2.3 [2.0–2.8]	2.0 [1.8–2.7]	0.232

Values are given as median [IQR]

A two-sided *p* value < 0.05 was considered statistically significant (in bold)

*RV* right ventricular, *PA* pulmonary artery, *PAP* PA pressure, *TAPSE* tricuspid annular plane systolic excursion, *sPAP*_*inv*_ invasively measured systolic PAP, *PAWP* PA wedge pressure, *DPG* diastolic pressure gradient, *TPG* transpulmonary gradient, *LAP* left atrial pressure, *RAP* right atrial pressure, *PP* pulse pressure, *PVR* pulmonary vascular resistance, *PAC *PA compliance, *CO* cardiac output, *CI* cardiac index

*DPG_neg_ and TPG_neg_ values were eliminated from final analysis

### RV–PA coupling and cardiovascular outcomes

**Fig. 1:**
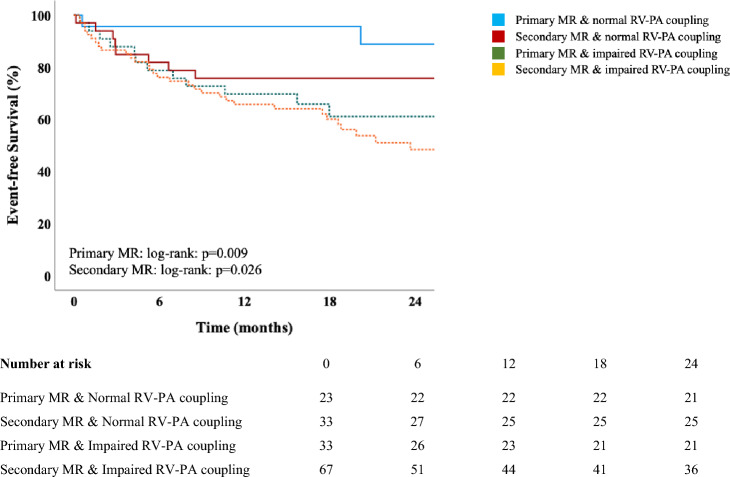
Kaplan–Meier curves demonstrating differences in time to the composite endpoint (heart failure hospitalization/death) stratified for MR etiology and RV–PA coupling in patients undergoing M-TEER

A total of 59 events (38 deaths, 21 HFH) occurred during follow-up (mean 19.5 ± 12.3 months). By Kaplan–Meier estimates, TAPSE/PASP ratio ≥ 0.36 was significantly associated with event-free survival at 2 years (log-rank: *p* < 0.001, Graphical Abstract). Similarly, when stratified for primary and secondary MR, impaired RV–PA coupling was found to have a significant impact on outcome (primary MR: log-rank: *p* = 0.009, secondary MR: log-rank: *p* = 0.026, Fig. 1). Cox-regression models demonstrating the association of RV–PA coupling with the primary endpoint of HFH and death are shown in Table 4 and Supplemental Table 2. After adjustment for the EuroSCORE II and NT-proBNP levels (Model B), TAPSE/PASP ratio < 0.36 on echocardiography was the only RV function parameter independently associated with outcome (adj.HR 2.65 [95% CI 1.32–5.34], *p* = 0.006). Findings were consistent across all multivariable models (Table 4 and Supplemental Table 2), also after excluding patients who underwent concomitant T-TEER (adj.HR 3.10 [95% CI 1.42–6.77], *p* = 0.004).

**Table 4:** Cox-regression analyses demonstrating the association of RV–PA coupling with the primary composite endpoint (HFH/death)

	Crude		Model A		Model B	
	HR (95% CI)	*p* value	Adj. HR (95% CI)	*p* value	Adj. HR (95% CI)	*p* value
TAPSE < 17 mm	2.27 (1.31–3.96)	**0.004**	2.01 (1.13–3.58)	**0.018**	1.62 (0.90–2.95)	0.111
RV FAC < 35%	1.60 (0.94–2.72)	0.081	1.62 (0.94–2.79)	0.081	1.29 (0.73–2.27)	0.379
S ‘ < 9.5 cm/s	2.20 (1.27–3.81)	**0.005**	1.99 (1.11–3.57)	**0.021**	1.68 (0.92–3.04)	0.090
RV free-lateral-wall strain > − 20%	2.18 (1.21–3.94)	**0.009**	2.08 (1.13–3.83)	**0.019**	1.73 (0.92–3.25)	0.089
RV global longitudinal strain > − 20%	2.32 (1.01–5.36)	**0.047**	1.88 (0.79–4.49)	0.157	1.74 (0.73–4.17)	0.214
TAPSE/PASP ratio < 0.274*	1.66 (0.99–2.78)	0.054	1.41 (0.80–2.47)	0.239	1.14 (0.64–2.02)	0.653
TAPSE/sPAP_inv_ ratio < 0.36	2.68 (1.28–5.60)	**0.009**	2.41 (1.13–5.14)	**0.023**	1.85 (0.86–3.96)	0.115
TAPSE/PASP ratio < 0.36	3.06 (1.58–5.91)	** < 0.001**	3.11 (1.55–6.26)	**0.001**	2.65 (1.32–5.34)	**0.006**

Model A: adjusted for the EuroSCORE II

Model B: adjusted for the EuroSCORE II and NT-proBNP levels (logarithmized)

A two-sided *p* value < 0.05 was considered statistically significant (in bold)

*RV–PA* right ventricular-to-pulmonary artery, *HFH* heart failure hospitalization, *NT-proBNP* N-terminal prohormone of brain natriuretic peptide, *HR* hazard ratio, *CI* confidence interval, *Adj*. adjusted, *TAPSE* tricuspid annular plane systolic excursion, *RV* *FAC* RV fractional area change, *S*’ tissue Doppler derived systolic movement of the RV lateral wall, *PASP* PA systolic pressure, *sPAP*_*inv*_ invasively measured systolic PAP

*Cut-off according to Karam N et al. JACC Cardiovasc Imaging 2021

### Reverse RV remodeling

**Fig. 2:**
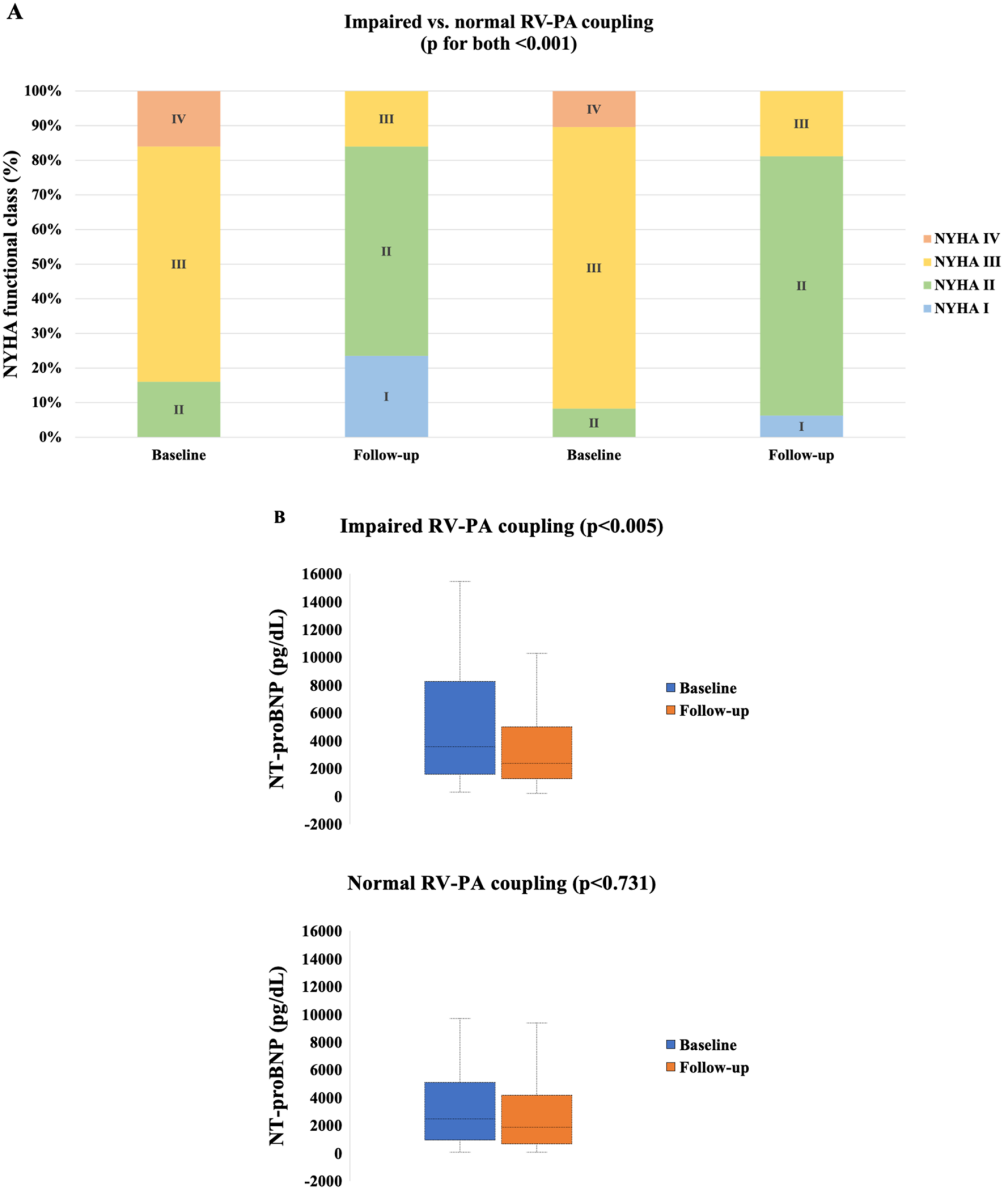
Changes in **A** NYHA functional class, and **B** NT-proBNP at baseline and 1-year follow-up after M-TEER stratified for baseline RV–PA coupling (*n* = 129)

Comprehensive follow-up data were available in 129 (83%) patients following M-TEER (mean 15.7 ± 11.2 months), of whom 28 (22%) underwent concomitant T-TEER. NYHA functional capacity significantly improved after M-TEER in both patients with impaired (NYHA grade ≥ III: 84–16%, p < 0.001) and normal RV–PA coupling at baseline (NYHA grade ≥ III: 92–19%, *p* < 0.001, Fig. 2A). NT-proBNP levels and Creatinine decreased significantly in individuals with impaired coupling (3537 to 2431 pg/mL, Fig. 2B, 1.3–1.2 mg/dL, *p* for both < 0.044), but remained unchanged in the group with preserved TAPSE/PASP ratio (2438–1805 pg/mL, 1.2–1.3 mg/dL, *p* for both > 0.366).

**Fig. 3:**
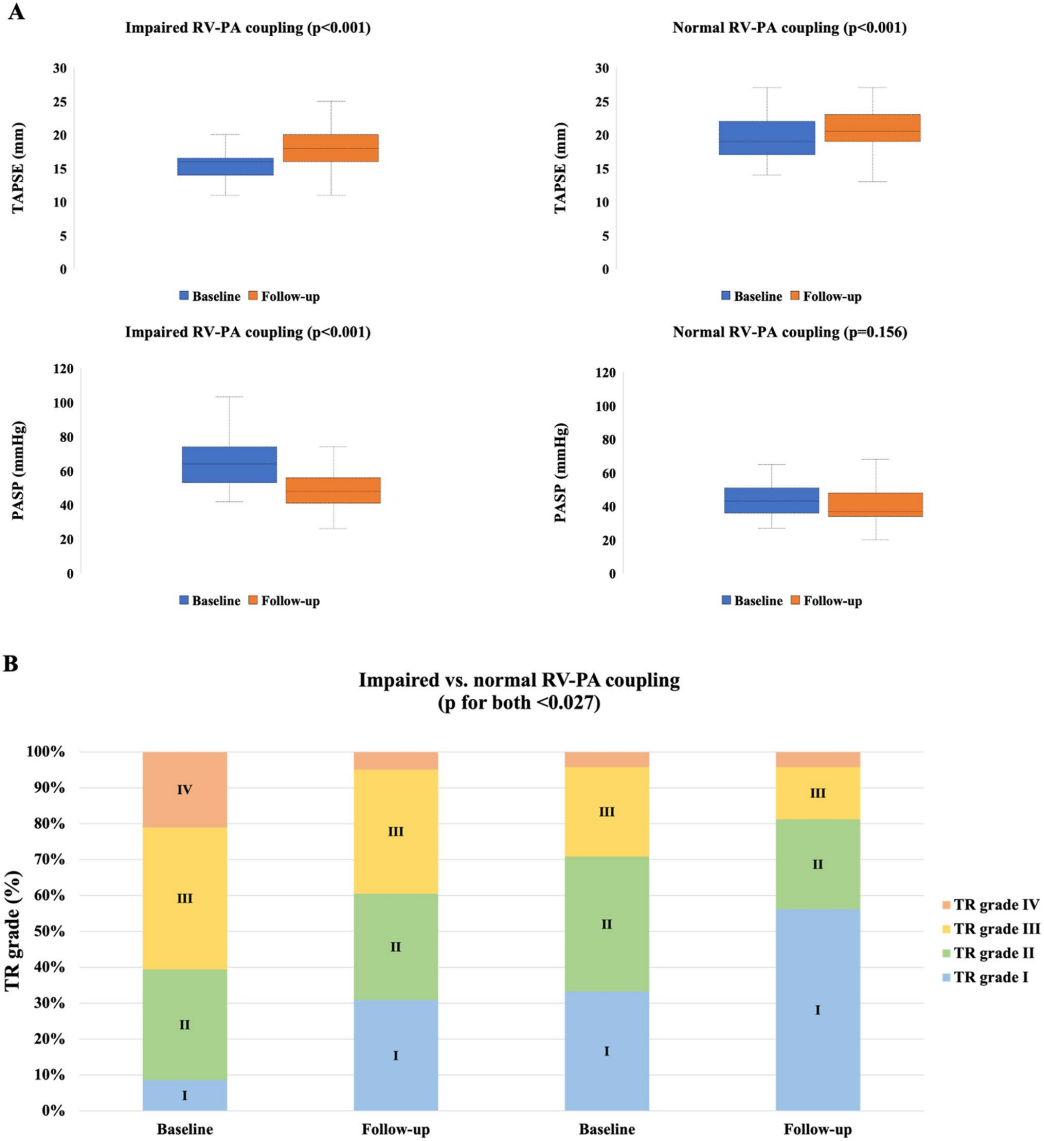
Changes in **A** TAPSE as well as PASP, and **B** TR grade at baseline and 1-year follow-up after M-TEER stratified for baseline RV–PA coupling (*n* = 129)

Following M-TEER, RV–PA coupling improved globally (TAPSE: ∆ + 3 mm, PASP: ∆ − 10 mmHg, *p* for both < 0.001, Fig. 3A), both in patients with primary MR (TAPSE: ∆ + 2 mm, PASP: ∆ − 12 mmHg, *p* for both < 0.001) and secondary MR (TAPSE: ∆ + 2.5 mm, PASP: ∆ − 9 mmHg, *p* for both < 0.001). However, changes were more profound in the group with impaired coupling (TAPSE: ∆ + 2 mm, PASP: ∆ − 13 mmHg, *p* for both < 0.001), when compared to normal coupling at baseline (TAPSE: ∆ + 1.5 mm, *p* < 0.001, PASP: ∆ − 6 mmHg, *p* = 0.156). Supplemental Fig. 1 shows changes in TAPSE/PASP ratio stratified for post-procedural MR. Similarly, at follow-up, RV size decreased only in patients with impaired coupling, but did not change in patients with normal coupling (RV end-diastolic diameter: ∆ − 5.5 mm vs. ∆ − 3 mm, *p* < 0.001 and *p* = 0.113, respectively). With regards to TR, following M-TEER, in both groups, a significant reduction of TR severity could be achieved (impaired vs. normal coupling, grade ≥ II: 91–69% and 67–44%, *p* for both < 0.027, Fig. 3B). Of note, these changes remained consistent among patients with impaired coupling at baseline and in absence of concomitant T-TEER (TR grade ≥ II: 88–65%, *p* = 0.002), but not in patients with preserved TAPSE/PASP ratio (TR grade ≥ II: 61–39%, *p* = 0.112).

## Discussion

In this comprehensive analysis of M-TEER patients, we observed that (1) TAPSE/PASP ratio was the only RV function parameter significantly associated with outcome in primary as well as secondary MR; (2) reverse RV remodeling, alongside with a reduction of TR severity, was evident in the majority of individuals following M-TEER; however, (3) these changes were only significant in patients with impaired RV–PA coupling at baseline.

M-TEER has increasingly been performed in recent years; however, pre-procedural risk assessment remains challenging. So far, accepted prognostic factors for mid- and long-term prognosis include pulmonary hypertension [21], renal failure [22], poor LVEF, and post-procedural residual MR [23, 24]. Only a few studies have investigated the prognostic impact of RV function in patients undergoing M-TEER. Godino et al. [25] found that TAPSE < 16 mm and/or S’ < 10 cm/s was not associated with outcome up to 2 years after M-TEER. Conversely, registry data of 817 patients undergoing M-TEER showed that impaired RV–PA coupling, defined as TAPSE/PASP ratio ≤ 0.274, was associated with a twofold increased risk of death at 2 years. [8] In addition, Adamo et al. [26] reported that two-thirds of 501 patients improved their RV–PA coupling after M-TEER, which was also associated with better outcomes. Of note, all these studies solely included patients with secondary MR. In the present study, we were able to expand on these findings by demonstrating that TAPSE/PASP ratio on echocardiography—rather than derived from right heart catheterization or other echocardiographic RV indices—was independently associated with HFH and death, irrespective of the underlying MR etiology. Our findings are of particular interest, especially considering the recently published recommendation level IIa for M-TEER in the treatment of both primary and secondary MR [2]. Notably, the indication upgrade for primary MR is based on registry data [23] and results from randomized-controlled studies, such as the upcoming PRIMARY trial (NCT05051033), are highly anticipated to confirm or disprove present study results.

At follow-up, we observed a significant improvement in NYHA functional capacity in all patients undergoing M-TEER, irrespective of TAPSE/PASP ratio at baseline. Although rates for HFH/death at 1 year and 2 years were higher among individuals with impaired coupling (33% and 43%, respectively), when compared to normal coupling (16% and 18%, respectively), these patients show a significant improvement in RV function and size, alongside with a reduction in TR severity at follow-up, irrespective of concomitant T-TEER. Of note, these changes are in line with previously reported data [26] and remained consistent across both MR etiologies. Hence, patients with reduced TAPSE/PASP ratio at baseline show the best potential for optimization in terms of cardiac remodeling, provided that they survive the first years following M-TEER.

The present analysis has several strengths: prospectively collected data, comprehensive hemodynamic as well as RV assessment, mid- to long-term follow-up, and inclusion of primary MR. We were able to add important new information regarding the prognostic value of RV–PA coupling in significant MR and provide further insights into patient selection and reverse RV remodeling after M-TEER.

## Limitations

All data refer to the experience of a single center. However, this setting ensures consistency throughout the study period, including echocardiographic and hemodynamic assessment and post-processing workflows. Even though TTE studies were performed and interpreted by different investigators, inter-observer variability showed reasonable agreement. Of note, echocardiographic evaluation of PASP in the presence of significant TR must be interpreted with caution, thus may contributing to the only modest correlation between invasive and non-invasive measurements. In addition, baseline echocardiographic and hemodynamic assessment were performed at different time points throughout the study course. Hence, a respective bias with regards to variable fluid status cannot be completely ruled out. Finally, our study was not designed to compare prognostic implications of treatment alternatives, such as surgical MV treatment or medical therapy.

## Conclusion

In the present study, non-invasively assessed TAPSE/PASP ratio was the only RV function parameter independently associated with HFH and death in patients with significant MR. Individuals with impaired RV–PA coupling at baseline benefited from M-TEER, irrespective of the underlying MR etiology, especially in terms of clinical symptoms, reverse RV remodeling, and TR reduction 1 year after intervention.

## Supplementary Information

Below is the link to the electronic supplementary material.

Supplementary file1 (DOCX 103 KB)

## Abbreviations

HFH: Heart failure hospitalization
MR: Mitral regurgitation
PASP: Pulmonary artery systolic pressure
RV–PA: Right ventricle-to-pulmonary artery
TAPSE: Tricuspid annular plane systolic excursion
TR: Tricuspid regurgitation
M-TEER: Transcatheter edge-to-edge mitral valve repair
T-TEER: Transcatheter edge-to-edge tricuspid valve repair
