# Supplementary material for: Impact of right ventricular-to-pulmonary artery coupling on remodeling and outcome in patients undergoing transcatheter edge-to-edge mitral valve repair
Source: Clin Res Cardiol. 2023 Oct 23;114(2):156–67. doi: 10.1007/s00392-023-02318-w (PMC11839846; doi:10.1007/s00392-023-02318-w)
Supplement: Supplementary file 1 — Supplementary file1 (DOCX 103 KB) [file 392_2023_2318_MOESM1_ESM.docx]

**Supplemental Data**

**Supplemental Table 1.** Inter-observer variability of RV function parameters in a subset of 20 patients.

|  | **ICC (95% CI)** | ***P* value** |
| --- | --- | --- |
| TAPSE | 0.728 (0.409-0.888) | **<0.001** |
| RV FAC | 0.862 (0.670-0.946) | **<0.001** |
| S‘ | 0.704 (0.367-0.877) | **<0.001** |
| RV free-lateral-wall strain | 0.825 (0.593-0.930) | **<0.001** |
| RV global longitudinal strain | 0.827 (0.598-0.936) | **<0.001** |

Abbreviations: RV, right ventricular; ICC, intraclass correlation coefficient; CI, confidence interval; TAPSE, tricuspid annular plane systolic excursion; RV FAC, RV fractional area change; S’, tissue Doppler derived systolic movement of the RV lateral wall.

**Supplemental Table 2.** Cox-regression analyses demonstrating the association of RV-PA coupling with the primary composite endpoint (HFH/death). Model C: adjusted for all parameters with a significant influence at an univariable level using a stepwise approach (EuroSCORE II, NT-proBNP, RV end-diastolic diameter, mean PA pressure, MR postprocedural), excluding variables already incorporated in the EuroSCORE II.

|  | **HR** | **95% CI** | ***P* value** | **Adj. HR** | **95% CI** | ***P* value** |
| --- | --- | --- | --- | --- | --- | --- |
|  | **Univariable analysis** | | | **Multivariable analysis** | | |
| **Clinical parameters** |  |  |  |  |  |  |
| Age | 1.00 | 0.97-1.03 | 0.992 |  |  |  |
| Female sex | 0.67 | 0.38-1.12 | 0.120 |  |  |  |
| Body mass index | 1.01 | 0.96-1.07 | 0.642 |  |  |  |
| EuroSCORE II | 1.04 | 1.01-1.07 | **0.016** |  |  |  |
| NYHA functional class ≥ III | 1.03 | 0.47-2.26 | 0.947 |  |  |  |
| NT-proBNP (logarithmized) | 2.58 | 1.54-4.32 | **<0.001** |  |  |  |
| Creatinine | 1.37 | 1.10-1.67 | **0.004** |  |  |  |
| eGFR | 0.98 | 0.97-1.00 | **0.002** |  |  |  |
| **Co-morbidities** |  |  |  |  |  |  |
| Coronary artery disease | 1.42 | 0.84-2.38 | 0.190 |  |  |  |
| Myocardial infarction | 1.47 | 0.84-2.58 | 0.180 |  |  |  |
| Percutaneous coronary intervention | 1.32 | 0.78-2.21 | 0.302 |  |  |  |
| Coronary artery bypass grafting | 1.64 | 0.91-2.94 | 0.101 |  |  |  |
| Previous valve surgery | 1.01 | 0.51-2.00 | 0.970 |  |  |  |
| Previous pacemaker implantation | 1.85 | 1.10-3.09 | **0.020** |  |  |  |
| Atrial fibrillation | 1.45 | 0.81-2.58 | 0.211 |  |  |  |
| Arterial hypertension | 0.85 | 0.26-2.71 | 0.777 |  |  |  |
| Diabetes mellitus type II | 1.22 | 0.70-2.12 | 0.485 |  |  |  |
| Hyperlipidemia | 0.56 | 0.33-0.94 | **0.029** |  |  |  |
| Previous stroke | 1.54 | 0.61-3.84 | 0.360 |  |  |  |
| Cerebral artery disease | 1.59 | 0.81-3.14 | 0.181 |  |  |  |
| Peripheral artery disease | 2.55 | 1.34-4.84 | **0.004** |  |  |  |
| COPD | 1.36 | 0.76-2.45 | 0.303 |  |  |  |
| **Echocardiographic parameters** |  |  |  |  |  |  |
| LV end-diastolic diameter | 1.00 | 0.98-1.03 | 0.520 |  |  |  |
| RV end-diastolic diameter | 1.06 | 1.02-1.11 | **0.005** |  |  |  |
| Interventricular septum | 1.04 | 0.96-1.13 | 0.365 |  |  |  |
| Aorta ascendens | 0.97 | 0.90-1.04 | 0.339 |  |  |  |
| LV ejection fraction | 0.98 | 0.96-1.00 | **0.045** |  |  |  |
| RV fractional area change | 0.98 | 0.95-1.00 | **0.037** |  |  |  |
| S‘ | 0.85 | 0.75-0.97 | **0.017** |  |  |  |
| RV free-lateral-wall strain | 2.18 | 1.21-3.94 | **0.009** |  |  |  |
| RV global longitudinal strain | 0.93 | 0.87-0.98 | **0.007** |  |  |  |
| MR etiology | 1.63 | 0.92-2.90 | 0.095 |  |  |  |
| TR ≥ moderate | 1.17 | 0.66-2.06 | 0.592 |  |  |  |
| **Hemodynamic assessment** |  |  |  |  |  |  |
| Mean PAP | 1.03 | 1.00-1.07 | **0.037** |  |  |  |
| Systolic PAP | 1.03 | 1.00-1.05 | **0.035** |  |  |  |
| Diastolic PAP | 1.04 | 1.00-1.09 | **0.040** |  |  |  |
| PAWP mean | 1.02 | 0.98-1.07 | 0.265 |  |  |  |
| DPG | 1.08 | 0.95-1.22 | 0.243 |  |  |  |
| TPG | 1.04 | 0.99-1.10 | 0.129 |  |  |  |
| LAP mean | 1.04 | 1.00-1.09 | 0.051 |  |  |  |
| RAP mean | 1.04 | 0.99-1.10 | 0.085 |  |  |  |
| PP | 1.02 | 0.99-1.06 | 0.171 |  |  |  |
| PVR | 1.00 | 1.00-1.00 | 0.798 |  |  |  |
| PAC | 0.97 | 0.83-1.12 | 0.640 |  |  |  |
| CI | 1.07 | 0.68-1.66 | 0.778 |  |  |  |
| **Procedural data** |  |  |  |  |  |  |
| Concomitant T-TEER procedure | 1.30 | 0.71-2.39 | 0.393 |  |  |  |
| MR postprocedural | 2.47 | 1.72-3.55 | **<0.001** |  |  |  |
| MV meanPG postprocedural | 1.15 | 0.99-1.35 | 0.075 |  |  |  |
| **Binary definition of RV dysfunction** |  |  |  | **Model C: adjusted for above listed variables** | | |
| TAPSE <17mm | 2.27 | 1.31-3.96 | **0.004** | 1.46 | 0.60-3.56 | 0.404 |
| RV FAC <35% | 1.60 | 0.94-2.72 | 0.081 | 0.86 | 0.37-1.99 | 0.485 |
| S‘ <9.5cm/s | 2.20 | 1.27-3.81 | **0.005** | 1.24 | 0.55-2.79 | 0.612 |
| RV free-lateral-wall strain >-20% | 2.18 | 1.21-3.94 | **0.009** | 1.25 | 0.53-2.95 | 0.606 |
| RV global longitudinal strain >-20% | 2.32 | 1.01-5.36 | **0.047** | 2.04 | 0.62-6.70 | 0.242 |
| TAPSE/PASP ratio **<**0.274* | 1.66 | 0.99-2.78 | 0.054 | 0.98 | 0.42-2.29 | 0.958 |
| TAPSE/sPAP_inv_ ratio **<**0.36 | 2.68 | 1.28-5.60 | **0.009** | 1.58 | 0.57-4.33 | 0.377 |
| TAPSE/PASP ratio **<**0.36 | 3.06 | 1.58-5.91 | **<0.001** | 2.74 | 1.17-6.43 | **0.021** |

Abbreviations: RV-PA, right ventricular to pulmonary artery; HFH, heart failure hospitalization; NT-proBNP, N-terminal prohormone of brain natriuretic peptide; MR; mitral regurgitation; HR, hazard ratio; CI, confidence interval; Adj., adjusted; NYHA, New York Heart Association; eGFR, estimated glomerular filtration rate; COPD, chronic obstructive pulmonary disease; LV, left ventricular; RV FAC, RV fractional area change; S’, tissue Doppler derived systolic movement of the RV lateral wall; TR, tricuspid regurgitation; PAP, PA pressure; PAWP, PA wedge pressure; DPG, diastolic pressure gradient; TPG, transpulmonary gradient; LAP, left atrial pressure; RAP, right atrial pressure; PP, pulse pressure; PVR, pulmonary vascular resistance; PAC, PA compliance; CI, cardiac index; T-TEER, transcatheter edge-to-edge tricuspid valve repair; MV, mitral valve; meanPG, mean pressure gradient; TAPSE, tricuspid annular plane systolic excursion; PASP, PA systolic pressure; sPAP_inv_, invasively measured systolic PAP; *Cut-off according to Karam N et al., JACC Cardiovasc Imaging 2021.


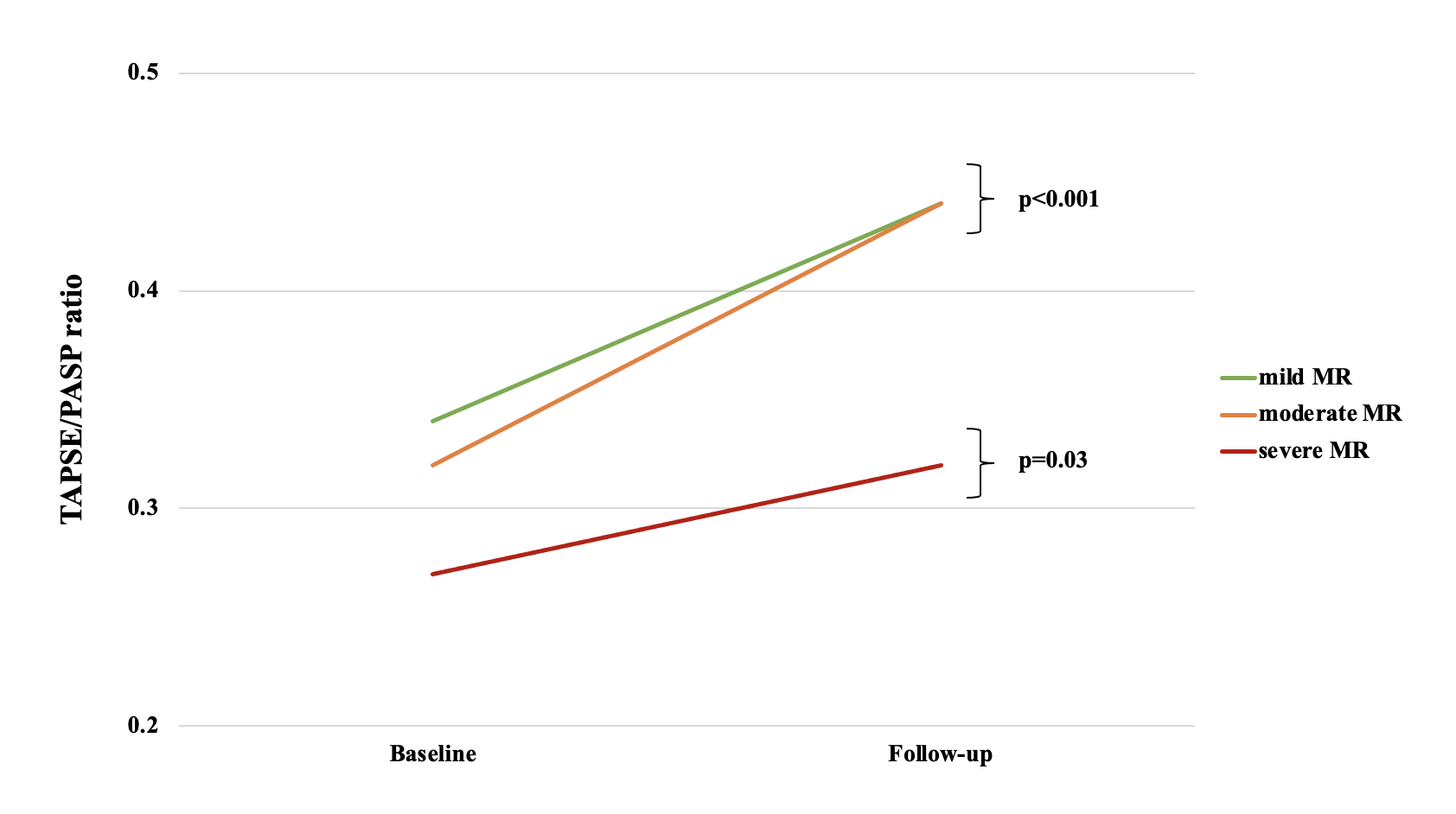
**Supplemental Figure 1.** Changes in TAPSE/PASP ratio at baseline and 1-year follow-up after M-TEER stratified for post-procedural MR (n=129).
